# Supplementary material for: Age-Related Alterations in Immune Contexture Are Associated with Aggressiveness in Rhabdomyosarcoma
Source: Cancers (Basel). 2019 Sep 17;11(9):1380. doi: 10.3390/cancers11091380 (PMC6770032; doi:10.3390/cancers11091380)
Supplement: Supplementary file 1 [file cancers-11-01380-s001.zip › cancers-582627-supplementary final/cancers-582627-supplement final.docx]

Supplemental Material

Age-Related Alterations in Immune Contexture are Associated with Aggressiveness in Rhabdomyosarcoma

Patrizia Gasparini, Orazio Fortunato, Loris De Cecco, Michela Casanova, Maria Federica Iannó, Andrea Carenzo, Giovanni Centonze, Massimo Milione, Paola Collini, Mattia Boeri,Matteo Dugo, Chiara Gargiuli, Mavis Mensah, Miriam Segale, Luca Bergamaschi, Stefano Chiaravalli, Maria Luisa Sensi, Maura Massimino, Gabriella Sozzi and Andrea Ferrari

**Table S1.** Kaplan Meier of RMS cohort.

|  | **Univariate HR** | **95% CI** | ***p*-Value** |  | **Multivariate HR** | **95% CI** | ***p*-Value** |
| --- | --- | --- | --- | --- | --- | --- | --- |
| **IRS**  IV vs. III vs. II vs I | 3.5 | 1.8–7.0 | 0.0004 |  | 3.0 | 1.5–6.1 | 0.0016 |
| **Histology**  Alveolar vs. Embrional | 2.8 | 1.2–6.8 | 0.0187 |  | 1.2 | 0.4–3.6 | 0.6925 |
| **Age**  ≥ 15 vs. < 15 years | 1.9 | 0.8–4.5 | 0.1458 |  | 1.8 | 0.6–4.8 | 0.2698 |

HR=Hazard Ratio; CI=Confidence interval; IRS= Intergroup Rhabdomyosarcoma Staging.

**Table S3.** 10 selected miRNA.

| **miRNA** | **Disease/Tumor** | **Involvement** |
| --- | --- | --- |
| miR-760 | Breast, Ovarian,Gastric, Lung, Colon | stem cell and metastasis |
| miR-223-3p | Melanoma, Kidney, blasdder, Lung, Prostate, Muscle, Heart failure | PAX4,PAX6 as targets |
| miR-432-5p | Lung, HCC, neuroblastoma, Ovarian, Muscle | inhibits myogenesis |
| miR-154-5p | Glioma, Lung, Breast, prostate, HCC, Leukemia | EMT, associated to estrogen |
| miR-376a-5p | Glioma, Ovarian, HCC, Prostate, melanoma |  |
| miR-493-5p | Melanoma, Breast, Gastric, Lung, Colon, Bladder | metastasis |
| miR-337-5p | Melanoma, Gastric, Neuroblastoma, Colon, Lung |  |
| miR-1185-5p |  | endothelial cells |
| miR-431-5p | Muscular atrophy, HCC, Lung, Glioma, Colon | age-related myogenic capacity |
| miR-382-5p | Ingantile hemangioma, Lung, Colon, Osteosarcoma |  |

**Table S5.** Top 20 genes deregulated in tumor and not in normal tissue.

| **GeneSymbol** | **RhoTumor** | **PValTumor** | **RhoNormal** | **PValNormal** |
| --- | --- | --- | --- | --- |
| **NOTCH2** | -0,575445072 | 2,67E-05 | 0,142857143 | 0,642828943 |
| **TOX2** | 0,505644811 | 0,000297189 | -0,087912088 | 0,77850131 |
| **CLUH** | 0,497177594 | 0,00038585 | -0,197802198 | 0,517155182 |
| **PLXNA4** | 0,492075554 | 0,000450231 | 0,17032967 | 0,578544469 |
| **STX1B** | 0,487190621 | 0,000520836 | 0,164835165 | 0,591181304 |
| **AHSA1** | -0,484802432 | 0,000558874 | 0,071428571 | 0,820631823 |
| **RPP21** | 0,482957013 | 0,000589968 | 0,054945055 | 0,863235584 |
| **BRSK2** | 0,468953539 | 0,000881723 | 0,17032967 | 0,578544469 |
| **GPR65** | 0,462548849 | 0,001054101 | 0,010989011 | 0,978311454 |
| **FGFR1** | -0,459183673 | 0,001156308 | -0,06043956 | 0,848988507 |
| **PRSS12** | 0,457555363 | 0,001208886 | -0,203296703 | 0,505254856 |
| **SLC38A2** | 0,45668693 | 0,001237796 | -0,021978022 | 0,949418437 |
| **ECSCR** | 0,451259227 | 0,001432961 | 0,148351648 | 0,62975814 |
| **C9orf152** | 0,448436822 | 0,001544965 | -0,131868132 | 0,669269054 |
| **SMIM29** | 0,448002605 | 0,001562875 | -0,010989011 | 0,978311454 |
| **ACAP2** | 0,445288754 | 0,001679079 | -0,027472527 | 0,934992303 |
| **SNAPC5** | 0,44279201 | 0,001792743 | -0,10989011 | 0,723248149 |
| **RAC2** | 0,439426835 | 0,001956801 | 0,005494505 | 0,992769769 |
| **HSPA14** | 0,439101172 | 0,001973367 | -0,010989011 | 0,978311454 |
| **OLFM3** | -0,438449848 | 0,002006875 | 0,197802198 | 0,517155182 |

**Table S6.** Recurrent genes observed in age-deregulated pathways.

| GENE | PATHWAYS INVOLVEMENT |
| --- | --- |
| SERPINE1 | TGFβ Signaling, EMT, Hypoxia, Complement, Xenobiotic Metabolism |
| IL6 | Apoptosis, EMT, Hypoxia, Complement, |
| TNFAIP3 | KRAS Signaling, EMT, Hypoxia, Complement, |
| PLAUR | KRAS Signaling, EMT, Hypoxia, Complement |
| ID2 | TGF β Signaling, KRAS Signaling, EMT, Xenobiotic Metabolism |
| BMP2 | TGF β Signaling, Apoptosis, KRAS Signaling |
| IRF1 | Apoptosis, Complement |

**Table S7.** Antibody sources and dilutions.

| **Antigens** | **Dilution** | **Code Number** | **Clone** | **Source** |
| --- | --- | --- | --- | --- |
| **CD3 (P)** | 1/400 | A0452 | Polyclonal | Dako,Agilent, Denmark |
| **CD4 (M)** | 1/300 | M7310 | 4B12 | Dako,Agilent, Denmark |
| **CD8 (M)** | 1/20 | M7103 | C8/144B | Dako,Agilent, Denmark |
| **CD68 (M)** | 1/3000 | M0814 | KP1 | Dako,Agilent, Denmark |
| **Granzyme B (M)** | 1/50 | M7235 | GrB-7 | Dako,Agilent, Denmark |
| **Myeloperoxidase (P)** | 1/4000 | A0398 | Polyclonal | Dako,Agilent, Denmark |
| **HLA-1 (M)** | 1/4000 | ab70328 | EMR8-5 | Abcam |
| **HLA-DR(M)** | 1/500 | MS-133-P0 | LN3 | Thermo fisher scientific |

M: monoclonal; P: polyclonal.


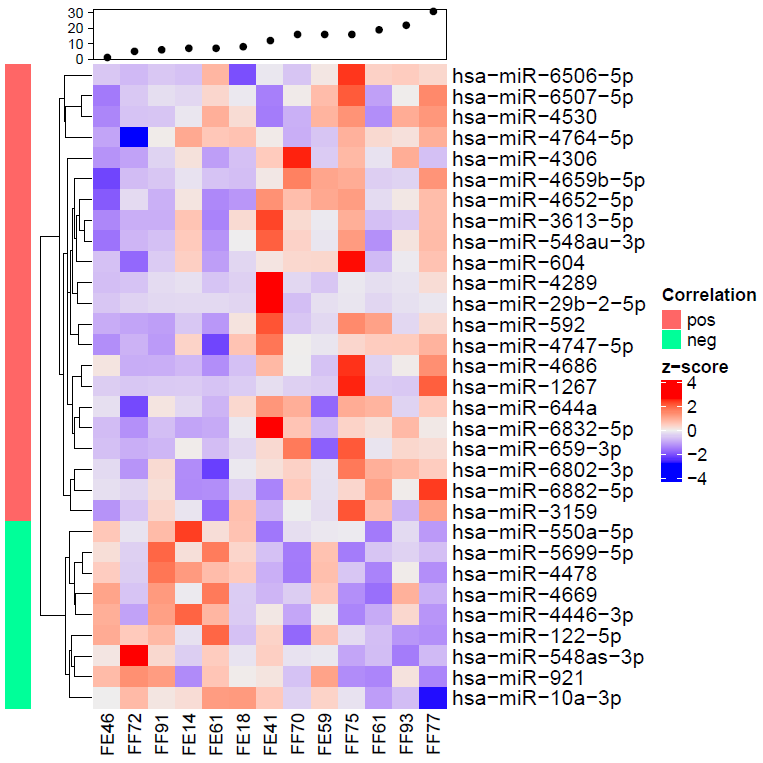


**Figure S1.** miRNA modulation in non neoplastic tissues. Heatmap showing the expression of the age-correlated miRNAs in non neoplastic tissues according to Spearman’s rank correlation test sorted by age (the black dots above the heatmap). MiRNAs corresponding to the red vertical bar are positively correlated and their expression may increase with age, while those associated to the green bar are negatively correlated (n = 13).

**Figure S2.** Integrated analysisi in non neoplastic tissues. GSEA analysis revealed pathways enrichment in non neoplastic tissues according to mir-223 (**A**) and mir-431 (**B**).


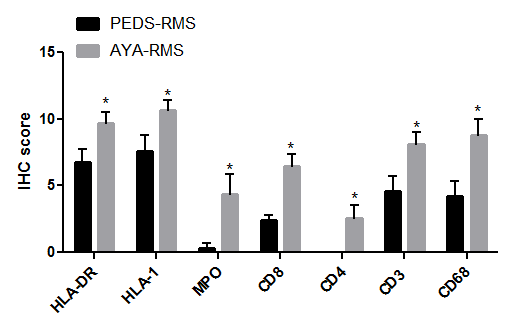


**Figure S3.** Immune cells quantification. Bar graphs show immune cells modulation in AYA-RMS compared to PEDS-RMS (n = 10). Data are expressed as mean ± SEM. **p* < 0.05.
